# Nanotechnologies for Reactive Oxygen Species“Turn-On” Detection

**DOI:** 10.3389/fbioe.2021.780032

**Published:** 2021-11-03

**Authors:** Hongfei Jiang, Qian Lin, Zongjiang Yu, Chao Wang, Renshuai Zhang

**Affiliations:** ^1^ Cancer Institute, The Affiliated Hospital of Qingdao University and Qingdao Cancer Institute, Qingdao, China; ^2^ Key Laboratory of Biobased Materials, Qingdao Institute of Bioenergy and Bioprocess Technology, Chinese Academy of Sciences, Qingdao, China

**Keywords:** reactive oxygen species, ROS nanotechnology, ROS turn-on detection, detection method, sensor

## Abstract

Reactive oxygen species (ROS) encompasses a collection of complicated chemical entities characterized by individually specific biological reactivities and physicochemical properties. ROS detection is attracting tremendous attention. The reaction-based nanomaterials for ROS “turn-on” sensing represent novel and efficient tools for ROS detection. These nanomaterials have the advantages of high sensitivity, real-time sensing ability, and almost infinite contrast against background. This review focuses on appraising nanotechnologies with the ROS “turn-on” detection mechanism coupled with the ability for broad biological applications. In this review, we highlighted the weaknesses and advantages in prior sensor studies and raised some guidelines for the development of future nanoprobes.

## Introduction

Reactive oxygen species (ROS) is the group of reactive anionic and neutral small molecules which are produced within many cell types. It mainly includes singlet oxygen (^1^O_2_), superoxide anion (O_2_^•−^), hydroxyl radical (^•^OH), and hydrogen peroxide (H_2_O_2_) (Yang et al., 2019). ROS has been confirmed to play a significant role in regulating numerous physiological functions of living organisms. However, ROS overproduction leads to oxidative stress and results in oxidative damage to a number of biomolecules including lipids, nucleic acids, proteins, and carbohydrates (Mattila et al., 2015), which is implicated in various diseases such as cancer, cardiovascular disease, diabetes mellitus, and aging (Valko et al., 2007; Winyard et al., 2011). Therefore, to improve the understanding of redox biology, the source and the stimulation of ROS generation, along with the consequences, we need to monitor and quantify ROS in cells, tissues, and whole organisms. Furthermore, the accurate species needs to be identified for each biological condition to fully understand redox biology.

**FIGURE 1:**
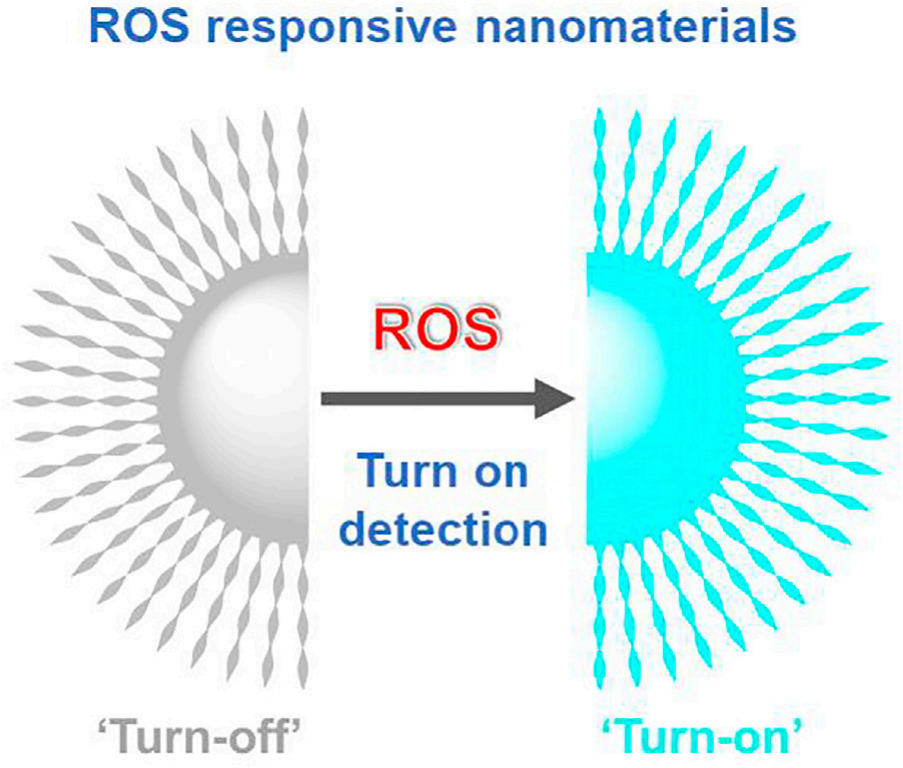
Nanotechnologies for ROS “turn-on” detection.

Joint efforts have been made by chemists and biologists to monitor the locations and concentrations of these highly aggressive species with very short lifetime. Thanks to these precise ROS detection methods, remarkable progress has been witnessed in unveiling the relevant biological mechanisms and uncovering the apparently paradoxical roles of distinct ROS in human health and disease. Small molecule fluorescent probes, especially reaction-based “turn-on” fluorescent probes, are generally useful owing to their high levels of sensitivity and capability to be applied in temporal and spatial sampling for *in vivo* and live cell imaging (Wu et al., 2019; Wang et al., 2020; Zhang et al., 2021). Alternatively, great varieties of nanomaterials with peculiar ROS-regulating abilities have been fabricated to support ROS science in the aspects of ROS generation, depletion, transition, and detection (Zhou Z. et al., 2016); these nanotechnologies finally benefit the ROS-based therapeutic outcomes (Yang et al., 2019; Yu et al., 2020; Zhang et al., 2021; Zheng et al., 2021). Nanoparticles exhibit tunable properties in size, shape, and function that make them flexible in process and product control for a wide range of applications (He et al., 2021; Yang et al., 2021; Yin et al., 2021). Using nanoparticles as probes, probe vectors, and compartmentalization agents for ROS detection has become more and more popular. Rationally designed nanotechnologies for ROS “turn-on” detection (Figure 1) are expected to possess the advantages of tunable functional group control, low cellular toxicity, high levels of sensitivity, and in particular, the capability of temporal and spatial sampling for *in vivo* and living cell imaging (Wu et al., 2019). It is worth mentioning that the benefit of “turn-on” over “turn-off” sensors is that they have almost infinite contrast against background. In principle, nanomaterials for ROS “turn-on” detection include carbon dots, silica nanoparticles, metal−organic framework (MOF), and nanoflakes. Herein, a selection of state-of-the-art nanomaterials for “turn-on” sensing of ROS with demonstrated promising application in biological systems is reviewed. We will appraise in detail these nanotechnologies with exactly demonstrated reaction mechanisms to help researchers choose suitable nanoprobes or inspire the development of future nanotechnologies.

## Detection of H_2_O_2_

**FIGURE 2:**
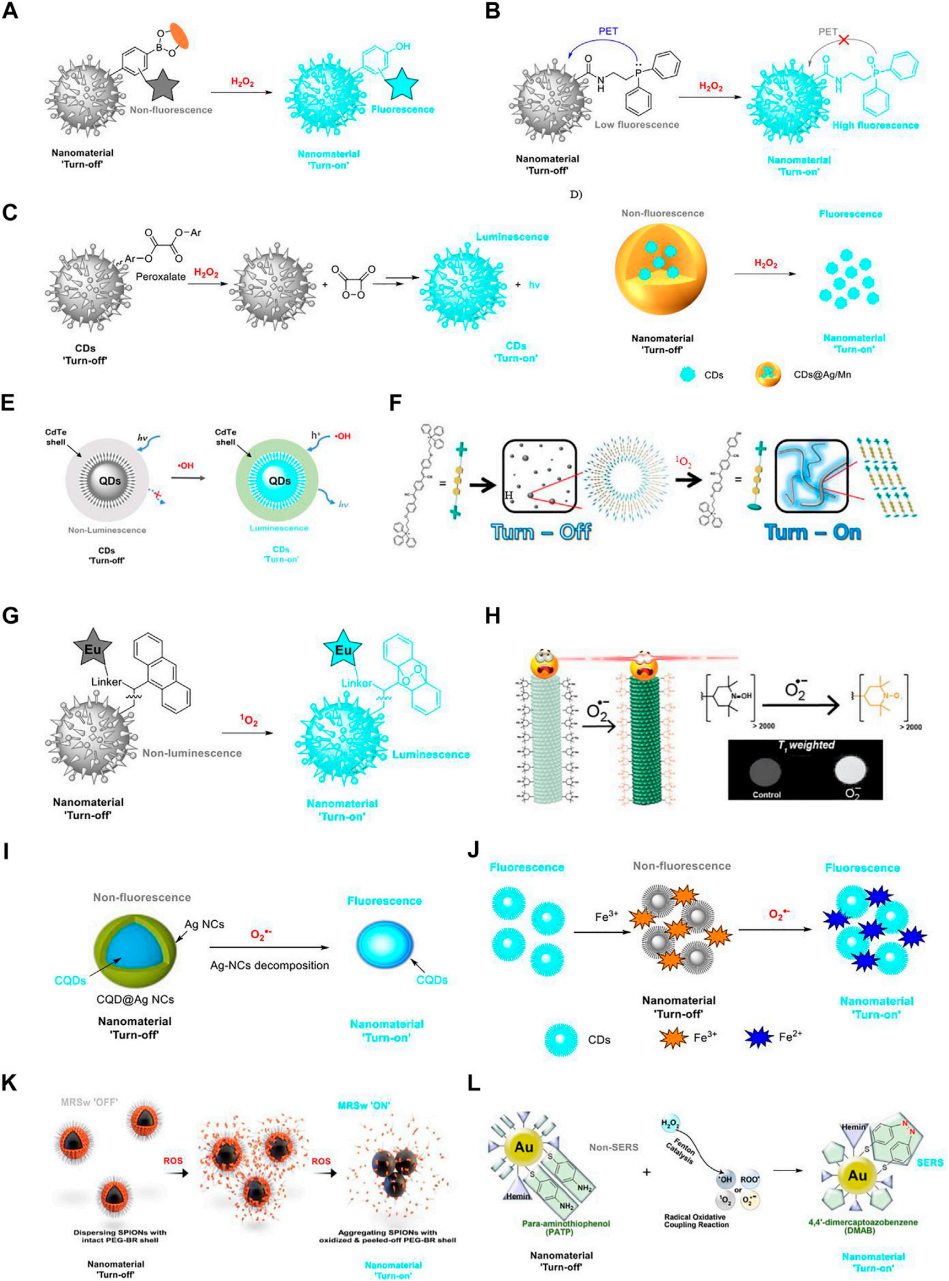
Nanotechnologies for ROS detection with a “turn-on” mechanism. **(A)** Boronic acid/boronate ester-based nanomaterials for H_2_O_2_ “turn-on” detection; **(B)** carbon dot-based fluorescence “turn-on” probe for H_2_O_2_ detection with a PET mechanism; **(C)** peroxalate-functionalized carbon nanodots as near-infrared chemiluminescent nanomaterial for H_2_O_2_ “turn-on” detection; **(D)** Ag -and Mn-based nanomaterials for H_2_O_2_ “turn-on” detection; **(E)** semiconductor quantum dots as “turn-on” luminescent probes for real-time detection of •OH; **(F)** triphenylphosphonium-based self-assembled nanomaterial for ^1^O_2_ “turn-on” detection; **(G)** “turn-on” detection of ^1^O_2_ using a nanostructured porous silicon microcavity through photonic luminescence enhancement strategy; **(H)** metal-free magnetic resonance imaging (MRI) tool for O_2_^•−^ “turn-on” detection (copyright 2018 American Chemical Society); **(I)** schematic illustrations of fluorescence “turn-on” detection of O2^•−^ based on CQD@Ag NCs (copyright 2017 Springer); **(J)** illustration of CDs-Fe^3+^ for the detection of O_2_^•−^; **(K)** PEG-BR@SPIONs as the biosensor with a magnetic relaxation switching-based mechanism for ROS “turn-on” detection; **(L)** Au-PATP-Hemin nanoprobe for ROS “turn-on” detection (copyright 2018 American Chemical Society).

H_2_O_2_ is a reactive species among ROS and is closely related to various physiological processes, such as cell proliferation, apoptosis, differentiation, and other signal transmissions. Therefore, the accumulation of excessive H_2_O_2_ has been implicated in many diseases. Thus, it is of great significance in monitoring the concentration of H_2_O_2_ in a physiological environment. As H_2_O_2_ is the most studied species of ROS, numerous mapping tools including radiative recombination mechanism and “dark” biological processes have been developed for H_2_O_2_ detection (Wu et al., 2019). Inspired by the successful development of boronic acid-based molecular fluorescent probes for H_2_O_2_ detection with a “turn-on” mechanism (Bull et al., 2013), several boronic acid functionalized fluorescent nanoprobes have been designed for “turn-on” sensing of H_2_O_2_. This design relies on the formation of the non-fluorescence boronic acid/boronate ester, which contains an electrophilic boron center; it reacts rapidly with H_2_O_2_, resulting in accelerated oxidative cleavage to afford the corresponding phenol to “turn on” the fluorescence (Figure 2A). Depending on this mechanism, Wu and co-workers developed a fluorescence resonance energy transfer (FRET)-based ratiometric fluorescent probe for the detection of H_2_O_2_. This nanoparticle uses carbon dots as the energy donor and carrier. The small size (∼4 nm) and good *in vivo* utility of this nanoparticle may support its eventual application in clinics (limit of detection (LOD) = 0.5 μM). Higher selectivity has been achieved for the nanoparticle for the detection of H_2_O_2_ over other ROS and biologically relevant species (Wu et al., 2014). Zhao’s group attached boronate ester to the surface of functional mesoporous silica nanoparticles (MSNPs) for “turn-on” detection of H_2_O_2_ (LOD = 3.33 μM). Moreover, they fabricated a H_2_O_2_-triggered drug release system for heart failure therapy. This system exhibits the potential for different variants of heart failure models to target theranostic treatment (Tan et al., 2017). The first MOF for H_2_O_2_ sensing have been designed by Sk and co-workers. Different from the previous two nanoparticles, the MOF directly uses boronic acid as a functional group attached to a Zr(IV) MOF for “turn-on” sensing of H_2_O_2_ in live cells (LOD = 0.015 μM). However, it also has moderate response to some other ROS and biologically relevant species, indicating that the selectivity of this MOF material toward H_2_O_2_ is a major defect (Sk et al., 2018). Then a boronic acid-functionalized 3D indium MOF was fabricated for H_2_O_2_ detection. The MOF exhibits an improved selectivity for H_2_O_2_ with an LOD of 420 nM (Jiang et al., 2021). In general, boronic acid-functionalized nanomaterials are easier for fabrication, while boronate ester nanomaterials possess higher selectivity for H_2_O_2_.

The carbon dot-based fluorescence “turn-on” probe for H_2_O_2_ with a photo-induced electron transfer (PET) mechanism was fabricated by Zhang’s group. In this nanoprobe, diphenylphosphine moiety is covalently attached to the surface of the carbon dot; they serve as the PET donor and acceptor, respectively. Subsequently, H_2_O_2_ can selectively oxidize the diphenylphosphine to produce the target oxide and prevent the PET mechanism; then the fluorescence will “turn on” (Figure 2B). The nanoprobe has a fast response to H_2_O_2_ with a LOD of 84 nM (Lan et al., 2015). Peroxalate-functionalized carbon nanodots are novel near-infrared chemiluminescent nanomaterials for H_2_O_2_ detection (LOD = 5 nM). Nanointegration of near-infrared carbon nanodots and peroxalate (P-CDs) with amphiphilic triblock copolymer as bridge can serve as “turn-on” sensors for the detection and imaging of H_2_O_2_ (Figure 2C). The high efficiency and large penetration depth of near-infrared photons of P-CDs make this strategy a good choice for bioimaging of H_2_O_2_ *in vitro* and *in vivo* (Shen et al., 2020).

Ag-based nanomaterials have broad application for H_2_O_2_ detection. In these designs, Ag materials normally act as a shell and serve as efficient quenchers, while H_2_O_2_ can prevent the Ag material-mediated quenching mechanism and fulfill the “turn-on” detection of H_2_O_2_ (Figure 2D). Chu’s group utilized DNA-templated Ag nanoparticles (DNA-AgNPs) coupled with NaYF4:Yb/Tm@NaYF4 shell upconversion nanoparticles (UCNPs) for the detection of H_2_O_2_ (LOD = 1.08 μM), in which, UCNPs and DNA-AgNPs serve as donors and quenchers, respectively. This design results in luminescence quenching of UCNPs using DNA-AgNPs by luminescence resonance energy transfer (LRET). Upon H_2_O_2_ introduction, AgNPs can be converted to Ag^+^, leading to the inhibition of the LRET process and inducing the recovery of upconversion luminescence (Wu et al., 2016). In this way, graphene quantum dots (QDs) adopted with the silver shell (GQD@Ag, LOD = 2 μM) (Kong et al., 2017), nitrogen-doped carbon QDs coated with silver nanoparticles (N-CQD/AgNPs, LOD = 4.7 μM) (Walekar et al., 2017), and a novel nanocluster-mediated chemical information processing system (CIPS, LOD information unavailable) (Zhao et al., 2018) have been designed and applied in selective H_2_O_2_ sensing with a “turn-on” mechanism.

Alternatively, the Mn-mediated nanotechnologies share a similar mechanism with Ag-mediated nanomaterials for H_2_O_2_ “turn-on” detection but have higher selectivity (Figure 2D). In this process, MnO_2_ nanosheets serve as a quencher but can be oxidized by H_2_O_2_ to fulfill the “turn-on” sensing of H_2_O_2_. Depending on this design, Yuan and co-workers fabricated manganese dioxide (MnO_2_)-nanosheet-modified UCNPs for rapid detection of H_2_O_2_ (LOD = 0.9 μM). The MnO_2_ nanosheets on the surface of UCNPs serve as the quencher. Fluorescence of UCNPs will be recovered after the addition of H_2_O_2_, which can reduce MnO_2_ to Mn^2+^ and destroy the structure of the MnO_2_ quencher (Yuan et al., 2015). Following this design, Lei and Liu’s group developed a carbon dot-MnO_2_ probe (LOD = 0.87 μM) and a three-in-one stimulus-responsive nanoplatform (Au@MnO2@Raman reporter, LOD = 6–7 μM), respectively, for H_2_O_2_ sensing with relatively improved selectivity or sensitivity (Ning et al., 2020; Zhang et al., 2020). While the Ag and Mn-mediated nanomaterials are utilized for H_2_O_2_ sensing in solutions, the biocompatibility of such structures is still questionable.

## Detection of Hydroxyl Radical (^•^OH)

^•^OH, the result of the homolytic cleavage of water (H_2_O → ^•^OH + ^•^H), is the most deleterious and reactive species of ROS. The general reactivity of the main ROS in biological systems decreases in the order of ^•^OH > ^1^O_2_ > H_2_O_2_ > O_2_^•−^ (Mattila et al., 2015). ^•^OH can destroy a number of biomolecules including proteins, lipids, and DNA, so as to induce numerous oxidative stress-related diseases. However, at present time, the detailed function of ^•^OH has seldom been demonstrated owing to the extremely high reactivity and short lifetime (Bai et al., 2019). Therefore, real-time sensing of ^•^OH in biological samples is of great importance. The use of semiconductor QDs as “turn-on” luminescent probes for real-time detection of ^•^OH has been developed (LOD = 0.3 μM) (Figure 2E). In this design, metal citrate complexes are adopted on the surfaces of QDs and can act as electron donors, injecting electrons into the lowest unoccupied molecular orbital (LUMO) of the QDs. Interestingly, only ^•^OH can inject holes into the highest occupied molecular orbital (HOMO) of the QDs. Consequently, the produced electron–hole pairs could emit strong luminescence through electron–hole recombination. This nanotechnology is demonstrated to have an application in detecting the endogenous release of ^•^OH in living cells (Zhou W. et al., 2016). Alternatively, Yu’s group fabricated a polyhedral-AuPd nanoparticle-based dual-mode cytosensor (PH-AuPd NPs, LOD information unavailable) with a “turn-on”-enabled signal for ^•^OH sensing. In this strategy, tetramethylbenzidine (TMB) acting as a functional group on the cytosensor is oxidized to ox TMB, a colored product, and can be monitored through colorimetric analysis. Coupled with a rational design, the nanotechnology has been constructed as a convenient method for the sensitive detection of MCF-7 cells (LOD = 20 cells ml^−1^) (Wang H. et al., 2018).

## Detection of Singlet Oxygen

^1^O_2_ has raised vital interest recently as a result of its significance in both chemical and biological systems. ^1^O_2_ is the lowest excited electronic state of molecular oxygen but is recognized to be highly reactive. Studies have demonstrated that ^1^O_2_ is highly toxic and destroys key biological molecules including proteins, DNA, and unsaturated lipids. Depending on a triphenylphosphonium derivative, the self-assembled nanomaterial has been fabricated for ^1^O_2_ “turn-on” detection (LOD = 33–56 μM) (Figure 2F). However, these nanoparticles are responsive to both ^1^O_2_ and ClO^−^ (Choi et al., 2018). A strategy for “turn-on” detection of ^1^O_2_ using a nanostructured porous silicon microcavity (pSiMC, LOD = 37 nM) through photonic luminescence enhancements has been developed (Figure 2G). The pSiMC is modified with an Eu(III)-linker-anthracene complex. In the presence of ^1^O_2_, the formation of an endoperoxide in the 9,10 position of anthracene is confirmed. Changes in the anthracene moiety can result in changes to the emission of the Eu(III) ion so as to induce these nanoprobes to become luminescent (Jenie et al., 2017). Alternatively, a technique for electrical detection of ^1^O_2_ on the surface of silver nanoparticle film has been fabricated by Knoblauch and co-workers. Singlet oxygen sensor green (SOSG, LOD information unavailable) in this system functions as a crucial moiety for the fluorescence “turn-on” sensing process. The presence of ^1^O_2_ in this system can result in change in the SOSG fluorescence quantum yield, which permits a stronger energy transfer from the SOSG probe to a proximal silver nanoparticle island film located in the near-electric field of the probe. This induces an increase in the target electric current flow, allowing for the sensing of the ^1^O_2_ (Knoblauch et al., 2020).

## Detection of Superoxide (O_2_^•−^)

O_2_^•−^ is a by-product of ATP generation processes of the human body microenvironment, which plays a significant role in regulating biochemistry and organic pathology. Furthermore, exposure to excess O_2_^•−^ would oxidize organisms, biological membranes, and tissues and cause diseases such as hepatitis, cancer, and diabetes (Gorrini et al., 2013). Nanomaterials such as carbon dots (Liang et al., 2020; Yue et al., 2021), MOF (Das et al., 2019), and tobacco mosaic virus (TMV) nanoparticles (Dharmarwardana et al., 2018) have been fabricated as selective sensors for “turn-on” sensing of O_2_^•−^. Silver nanoparticle (Ag NP)-coated carbon quantum dot (CQD) core–shell-structured nanocomposites (CQD@Ag NCs) have been developed for fluorescent sensing of intracellular O_2_^•−^ (LOD = 0.3 μM) (Figure 2I). In CQD@Ag NCs, CQDs display a potent blue fluorescence; however, the fluorescence is quenched by Ag NPs. In the presence of O_2_^•−^, Ag NPs are oxide-etched, and the fluorescence of CQDs is recovered (Liang et al., 2020). Yue and co-workers fabricated similar carbon dots for O_2_^•−^ sensing while the quencher is Fe^3+^ (LOD = 25 pM). The addition of O_2_^•−^ can convert Fe^3+^ to Fe^2+^ and recover the fluorescence of carbon dots (Yue et al., 2021) (Figure 2J). It is reported that CQD@Ag NCs are successfully utilized in the imaging of O_2_^•−^ in MCF-7 cells; however, the biocompatibility of Liang’s carbon dots is questionable. The Gassensmith group managed to functionalize the surface of TMV (LOD information unavailable) nanoparticles with 4-hydroxy-tetramethylpiperidine at the protein tyrosine residue. The nanoparticles function as a metal-free magnetic resonance imaging (MRI) tool for O_2_^•−^ monitoring (Figure 2H). The mechanism of this strategy for O_2_^•−^ “turn-on” detection is that 4-hydroxy-tetramethylpiperidine can be oxidized to TEMPO, which has a different *T*_1_-weighted imaging. TMV nanoparticles can selectively respond to O_2_^•−^ without being affected by H_2_O_2_ and O_2_; however, no available data show whether other species of ROS, for example, the more reactive ^•^OH, can react with TMV nanoparticles (Dharmarwardana et al., 2018). However, a MOF material of the UiO family called Zr-UiO-66-NH-CH2-Py has been fabricated with a clear selectivity toward O_2_^•−^ over other ROS (LOD = 0.21 μM). Enhancement of the fluorescence response of the MOF upon stepwise addition of O_2_^•−^ has been recorded. The mechanism of the fluorescence “turn-on” procedure is recognized as follows: the structural collapse of the MOF in the presence of O_2_^•−^ can result in the release of the linker (2-((pyridin-4-ylmethyl)amino)terephthalic acid) with the enhancement of the fluorescence intensity of the system (Das et al., 2019).

## Detection of Combined Species of ROS

In some conditions, the evaluation of cellular or system total ROS provides helpful information on cell proliferation, metabolism, and tumor detection. Distinct from the design of nanotechnologies for selective sensing of specific species of ROS, these nanomaterials can detect the combined species of ROS by one platform. Several nanomaterials, including PEGylated bilirubin-coated superparamagnetic iron oxide nanoparticles (PEG-BR@SPIONs) (Lee et al., 2020), UCNPs-MoS_2_ nanoflakes (Wang F. et al., 2018), multifunctional theranostic nanoprobes (Au−Ag-HM) (Wang et al., 2021), para-aminothiophenol and hemin-decorated gold (Au-PATP-Hemin) nanoprobes (Cui et al., 2018), cyclotriphosphazene-doped graphene quantum dots (C-GQDs) (Xu et al., 2020), ROS-responsive microgel (Liu et al., 2018), and the ionic nanoparticles in a hydrogel microparticle (Liu et al., 2018), are designed under this context. Therein, PEG-BR@SPIONs and Au-PATP-Hemin nanoprobe are promising tools for “turn-on” detection of ROS with demonstrated mechanisms and have potentialities in biological applications. PEG-BR@SPIONs as a biosensor with a magnetic relaxation switching-based mechanism have been employed for whole-blood ROS sensing (LOD = 30–50 μM) (Figure 2K). The “turn-on” mechanism is actualized by the change of magnetic relaxation signal upon exposure to ROS. Furthermore, these ROS-responsive PEG-BR@SPIONs are utilized in a sepsis-mimetic clinical setting to directly monitor the total concentration of ROS in the blood samples through an explicit change in *T*_2_ magnetic relaxation signals and a “turn-on” signal of fluorescence. The design of a Au-PATP-Hemin nanoprobe is principled upon the discovery that PATP can react with ROS through a radical oxidative coupling mechanism to form 4,4′-dimercaptoazobenzene (DMAB), which can elicit potent characteristic surface-enhanced Raman scattering (SERS) signals at 1,142, 1,386, and 1,432 cm^−1^ and directly enable the detection of ROS through a hemin-catalyzed Fenton reaction (LOD = 26 pM) (Figure 2L). Simultaneous detection of five ROS species (^•^OH, ROO^•^, O_2_^•−^, ^1^O_2_, and H_2_O_2_) has been realized by the Au-PATP-Hemin nanoprobe. In two typical ROS-elevated mice models of allergic dermatitis and tumors, the Au-PATP-Hemin nanoprobe performed well in monitoring inflammation progression and tumor development in a sensitive and quantitative manner.

## Conclusion

In this short review, nanomaterials with the ability for ROS “turn-on” detection have been deciphered from the aspects of both nanotechnology and chemical reaction mechanisms. In general, “turn-on” nanotechnologies are powerful tools with a low detection limit, real-time sensing ability, and almost infinite contrast against background. Future studies for the design of ROS “turn-on” detection nanomaterials should make efforts to improve selectivity, detection limit, and biocompatibility. Another consideration is the accessibility of these nanomaterials. Reagents for the nanomaterial fabrication are commercially available or can be prepared in simple synthetic steps from commercially available building blocks, which will be greatly in vogue. Continuous efforts are poised to develop more powerful nanotechnologies in this promising field to shed light on critical information of ROS in biological systems.
